# Understanding Loneliness in Older Adults During the Pandemic: Predictors and Questionnaire Validation

**DOI:** 10.3390/diseases13020045

**Published:** 2025-02-04

**Authors:** Rahela Orlandini, Linda Lušić Kalcina, Vesna Antičević

**Affiliations:** 1 University Department of Health Studies, University of Split, 21000 Split, Croatia; vesna.anticevic@ozs.unist.hr; 2 School of Medicine, University of Split, 21000 Split, Croatia; llkalcina@ffst.hr; 3 Faculty of Humanities and Social Studies, University of Split, 21000 Split, Croatia

**Keywords:** personal traits, psychological traits, pandemic stressors, loneliness, mental health

## Abstract

Background/Objectives: The COVID-19 pandemic is behind us, but the experiences gained during its course can serve as a framework for preventive actions in future crises. The main objectives were to examine the predictors of loneliness in older adults during the pandemic and to explore the mediating effects of emotional stability between pandemic-specific stressors and loneliness. To achieve the set objectives, we developed a questionnaire to measure pandemic-specific stressors in older adults. Methods: A cross-sectional research design was used. A total of 578 participants of both genders (38.9% male, 61.1% female) aged 65 and above (M = 74.09, SD = 6.72) participated in this study. The self-reported measures used included the following: The Ten-Item Personality Inventory, The Revised Loneliness Scale, The Clinical Outcomes in Routine Evaluation, and The Pandemic-Specific Stressors Questionnaire for Older Adults. Results: Using exploratory factor analysis, two factors were extracted, providing evidence of face and convergent validity, together explaining 71.3% of the variance. The results of the confirmatory factor analysis indicated a good model fit. Hierarchical regression analysis indicated the greatest contribution of the psychological factors to loneliness in older adults during the pandemic, while marital status and pandemic-specific stressors had a minor but still significant impact. Mediation analysis revealed that emotional stability mediated the association between social distancing experiences and loneliness. Conclusions: In future global pandemics, it is necessary to pay full attention to psychological factors to preserve the mental health of older people. The newly-constructed questionnaire identifies pandemic-specific stressors in older adults, aiding their mitigation and easing recovery from the pandemic crisis.

## 1. Introduction

Loneliness, an unpleasant emotional state resulting from the gap between expected and actual relationships, was widespread during the pandemic [1]. Research distinguishes between social loneliness, which results from reduced social interactions, and emotional loneliness, which is linked to feelings of isolation due to a lack of meaningful connections [2]. Both forms of loneliness are associated with an increased risk of health deterioration, including higher risks of cardiovascular disease, diabetes, anxiety, depression, and dementia [3]. While aging does not inherently cause loneliness, older adults face numerous risk factors that can lead to it [4,5]. These include functional impairments, loss of supportive relationships, declining health, chronic illnesses, psychological issues, retirement, and global crises like epidemics and pandemics, which have significantly impacted human lives. Barakat et al. noted that the COVID-19 pandemic has heightened awareness of mental health, following a trend of previous outbreaks like MERS-CoV, Ebola, and Influenza, which disproportionately affected vulnerable groups, including older adults. Major disasters often lead to PTSD, major depression, and increased substance abuse, with fewer studies addressing other post-disaster psychiatric conditions [6]. Research on infectious disease outbreaks highlights the significant mental health impact on vulnerable populations, influenced by sociodemographic factors, personal resources, and mental health status [7]. Risk factors for distress include gender, age, economic factors, media exposure, and stigma, while protective factors include social support and leadership [8]. Experiences like quarantine and social distancing can have long-term mental health consequences [9].

The mental health impact of the COVID-19 pandemic on the general population re-mains debated. Although COVID-19 significantly affected mental well-being at both individual and collective levels [10], some studies suggest that the prevalence of mental health disorders did not increase during the pandemic [11]. However, public concerns were raised about the government’s response, as isolation measures were perceived to have a more profound effect on mental health, suicide rates, the economy, and vulnerable groups, such as the elderly, than the pandemic itself [12].

Research on older populations has shown that the COVID-19 pandemic has had a significant impact on the mental health and social well-being of older adults. Social isolation and loneliness have been linked to negative mental health outcomes, including anxiety, depression, neuroinflammation, and overall health decline [4,13,14,15,16,17]. The pandemic has created a “COVID-19 Social Connectivity Paradox”, where physical distancing measures intended to protect older adults simultaneously increase their risk of social isolation and loneliness [18]. During the COVID-19 pandemic, particular research interest focused on examining the impact of pandemic-specific stressors on loneliness in older adults. Research during the COVID-19 pandemic has shown loneliness to be a major issue for older adults world-wide. In Croatia, greater communication, including via technology, was linked to reduced social and family loneliness among older people [19]. A cross-country study found that those in hard-hit areas experienced more fear and loneliness, with variations across age and gender [20]. Another study across 27 European countries observed that while loneliness rose for some older adults, it decreased for others, and virtual communication could not replace face-to-face interaction [21]. Studies have shown that pandemic-specific stressors, such as social distancing measures and concerns about infection, are linked to increased loneliness among older adults [22,23,24]. It has also been found that, when enhancing capacities and resilience, it is important to prioritize women, older adults, individuals living alone, and people with chronic illnesses [25].

Research also indicates that while social isolation increased during lockdowns, loneliness levels remained relatively stable, affecting 28.6% of older adults, with social isolation impacting 31.2% [26]. Despite their overlap, loneliness and social isolation are distinct; loneliness is more closely associated with mental health issues, while social isolation is linked to poor self-rated health [27]. Given that these concepts overlap yet remain distinct, there was a need to create a new tool for measuring pandemic-specific stressors, including social isolation, specifically for older adults. At the beginning of the pandemic, many researchers were interested in measuring COVID-19-induced stress in people. In this regard, a number of questionnaires specifically designed to measure COVID-19-related stressors in the general population have been developed [28,29,30]. Most of them measured the severity of distress caused by the exposure to pandemic-specific stressors, the association of pandemic stressors with coping strategies, and symptoms of depression in urban and rural areas. However, a review of the literature did not find measurement instruments constructed specifically for measuring pandemic stressors in the older population at the time when current research was planned. To address this gap, we developed a new questionnaire tailored exclusively to older adults by identifying the most dominant stressors they faced during the pandemic. This methodology included responses directly identified by the target population, ensuring the tool’s relevance and applicability to the unique experiences of older adults. This tool considers that the older people in Croatia may face unique pandemic-specific stressors, such as specific family dynamics, challenges in accessing healthcare, and digital connectivity issues, which may differ from those in other cultural contexts. This localized tool aims to improve stakeholder engagement and its application in community and healthcare settings, ensuring its relevance and broader applicability within the Croatian context.

In addition to exposure to pandemic-specific stressors, previous research has examined the contribution of sociodemographic and psychological factors to loneliness in older adults during the pandemic. Several studies have indicated that during the COVID-19 pandemic, certain sociodemographic factors heightened the risk of loneliness among older adults [31]. These factors include age, living arrangements (such as living alone), residing in rural areas, widowhood, gender (being female), lower levels of education, and lower financial status [32,33,34,35,36,37]. Findings on the impact of age and gender on loneliness are inconsistent. While some studies found a higher likelihood of loneliness during the initial waves of COVID-19 in individuals under 60 compared to those aged 60 and above [38], others did not find such differences. Beam and Kim argue that being older alone does not necessarily lead to loneliness, with loneliness seemingly increasing with age only beyond 80 [32]. Similarly, regarding gender, findings vary. Certain studies concluded that being female posed a loneliness risk during the COVID-19 crisis [39,40], while others found no gender differences [37].

Previous research has highlighted that, alongside sociodemographic factors, psychological factors also contributed to loneliness in older adults during the COVID-19 pandemic. A review of the existing literature further revealed significant associations between loneliness and psychological distress across both the general population [41,42,43] and specifically among older adults [44]. The COVID-19 pandemic has significantly impacted older adults’ mental health and social well-being, with studies reporting increased loneliness and depression [24,45]. Factors associated with greater loneliness include anxiety symptoms [46] and financial strain [24]. Conversely, having a partner, emotional support, and being a person of colour were linked to lower loneliness levels [24]. Psychological resilience predicted lower loneliness and greater technology use, which mediated the relationship between resilience and loneliness [47]. Subjective age also played a role, with those feeling younger experiencing a weaker association between loneliness and psychiatric symptoms [48]. These findings highlight the complex interplay of factors affecting older adults’ well-being during the pandemic. Personality traits, particularly extraversion and neuroticism, were associated with loneliness levels [49]. Factors such as optimism, pessimism, and hopelessness also predicted loneliness scores [50]. However, certain factors were found to protect against loneliness during the pandemic. For example, satisfaction with social communication, emotional support, and having a spouse or partner were identified as factors that help reduce loneliness [22,24].

Based on the empirical evidence outlined, the primary objective of this study was to identify psychological and pandemic-related stressors, as well as demographic characteristics that predict loneliness in older adults during the pandemic. For the purpose of investigating the stated objective, we recognized the need to construct and validate a questionnaire specifically designed to assess pandemic-related stressors in the older population, which was the focus of this study. Consequently, the development and validation of the questionnaire served as a secondary objective, rather than a primary focus, of the study.

## 2. Materials and Methods

The methodological framework of this study adheres to the COSMIN framework [51], ensuring content validity through literature review, consultations with older adults, and expert assessments. Structural validity is supported by factor analyses (EFA and CFA), identifying and confirming two factors (social distancing and exposure to infection) explaining 71.3% of the variance, with good model fit indices. Internal consistency is confirmed with a Cronbach’s alpha of 0.877, though test–retest reliability could not be measured due to the cross-sectional design and difficulty accessing older adults during the pandemic. Construct validity was demonstrated through significant correlations with well-established instruments, including the UCLA, the CORE Outcome Measure, and the Loneliness Scale, providing evidence of convergent validity. Additionally, the study design restricted the assessment of responsiveness to changes over time. Nonetheless, interpretation of the findings was facilitated by comprehensive statistical analyses, adhering to the standards outlined in the COSMIN guidelines.

### 2.1. Participants

A cross-sectional survey-based research design with a non-randomized sampling method has been used in this research. The participants were the Croatian citizens of both genders, aged above 65 years, living in Split-Dalmatia County. To define “older adults”, we used the cut-off age of 65 years [52]. The sample comprised 578 participants (39% male) aged 74.09 ± 6.72 years, where twice as many participants lived in urban rather than in rural areas. Most of them were married (59.3%) or widowed (30.6%), while the others were divorced or never married. Regarding employment status, 82.9% of participants were retired, 12.5% never employed, and less than 5% worked either full-time or part-time. Additionally, more than a half of participants completed high (44.5%) or primary (23.2%) school, while the least of them had either an incomplete primary school (2.4%) or postgraduate education (1.4%) (Table 1). The exclusion criteria were an age < 65 and persons with dementia or acute psychosis.

The participants were recruited using the snowball sampling technique [53,54]. The snowball sampling technique was chosen to efficiently reach older adults, who often had limited mobility and were isolated from standard recruitment channels. This method used social networks to access participants quickly despite movement restrictions. Relying on trusted referrals fostered comfort and increased participation, while the approach was also resource-efficient, enabling data collection in a cost-effective, low-risk manner during the crisis. Sixty students of health studies identified at least six possible participants of both genders aged >65 from among their relatives and acquaintances. Then, those participants identified other persons who could be included in the research, and those persons indicated the next participants in the research and so on, “driving” the sampling process. This process continued until the predicted sample size (N = 578) was reached. Considering the known population in Split-Dalmatia County (75,451 inhabitants according to the population census of 2011), the sample size was calculated based on the Cochrane formula for continuous data: n_0_ = t^2^ × s^2^/d^2^ [55]. The value t represents the value for the selected alpha level in each tail. The value s is an estimate of standard deviation in the population (it is calculated when dividing the inclusive range of the scale (4-point scale of self-efficacy) with the number of standard deviations that would include all possible values in the range (3 standard deviations on each side of the scale), and then squaring this number. The value d is an acceptable margin of error for the mean, being an estimate (number of points on primary scale × acceptable margin of error). When defining the level of acceptable error as an alpha value at 1%, and setting the level of acceptable error at 3%, according to the calculation above, the required sample is 578 respondents.

Participants were contacted either directly or by telephone, depending on the epidemiological situation. When a direct interview was possible, students distributed questionnaires in envelopes to the participants. Participants filled out the questionnaires, sealed the envelopes, and returned them to the student who initially delivered them. Completing the questionnaire took an average of 20 min. Before filling out the questionnaire, students provided participants with an information letter explaining the research and a consent form to sign.

In cases where direct contact was not feasible, telephone interviews were conducted. Students read the information letter to the participants over the phone, and verbal agreement to participate was considered as consent. Personal data, including consent forms, were kept separate from the collected questionnaire data to ensure anonymity. Participant names and surnames were not collected, and additional measures were taken to safe-guard anonymity. Each set of questionnaires was placed in a separate envelope, and sealed envelopes were returned to the researchers via the students.

Exclusion criteria were addressed during participant screening. If a participant provided an affirmative answer to an exclusion factor, the survey was not conducted.

The study was conducted during the period from April to October 2021.

### 2.2. Measures

The following measures were used for collecting data and to examine research aims:

Ten-Item Personality Inventory—The Croatian version (TIPI). This 10-item short version of the Big-Five Inventory measures each of the five personality traits: Extraversion—E, Emotional Stability—ES, Agreeableness—A, Conscientiousness—C and Openness to Experience—O. Participants rated their personality traits on a seven-point Likert scale (1 = totally disagree to 7 = totally agree). Even though the TIPI showed slightly lower reliability levels of internal consistency in the original research studies (E = 0.68; A = 0.40; C = 0.50; ES = 0.73; and OE = 0.45 [56], the test–retest reliability and validity levels satisfy what enabled this measure to be used. For the Croatian version of the questionnaire, the reliability coefficients are as follows: E = 0.36; A = 0.13; C = 0.38; ES = 0.46; OE = 0.41) [57]. Given that the reliability coefficients in this study are low, we included only the scale with the highest reliability, i.e., emotional stability (ES = 0.46), in the analysis. The inclusion of the Ten-Item Personality Inventory (TIPI) and its Emotional Stability subscale in this study is warranted despite the relatively low reliability coefficients. This decision is justified by the study’s focus on older adults during the pandemic, where the need for brevity in assessments was a critical consideration. As noted by Saucier [58], the TIPI provides an efficient solution in studies where minimizing survey length is a priority. Its non-redundant items also help reduce participant fatigue [59], which can be particularly important in studies involving older adults. Despite its lower Cronbach’s alpha, the TIPI is widely used in exploratory research, as it maximizes content validity while minimizing administration time [56,60]. Given these considerations, including the Emotional Stability scale as part of the TIPI is a reasonable choice for this study.

The revised UCLA Loneliness Scale—The Croatian short-form version. A 7-item short version of the UCLA Loneliness Scale measures loneliness as a general unidimensional construct defined as an unpleasant experience caused by the inability of an individual to meet his/her needs for intimacy, love, and affiliation. Participants rate each item on a 5-point Likert scale, from 1 (does not apply to me at all) to 5 (totally applies to me). The total score is a sum of ratings of all items divided by the number of items [61]. In both international and Croatian samples, the unidimensional structure of the scale and its high reliability were confirmed. In the Croatian sample, the Cronbach alpha coefficient was 0.84 [62].

The Clinical Outcomes in Routine Evaluation Outcome Measure (CORE-OM). The Croatian version is a 34-item self-reported measure of psychological distress. Items refer to four dimensions: Subjective Well-being (4 items), Problems/Symptoms (12 items), Daily Functioning (12 items), and Risky Behaviours (6 items). These domains are not separate linear factors but different areas of expression of distress and dysfunction. The participants assess how often they felt as described during the past two weeks (0—never, 1—very rarely, 2—sometimes, 3—often, 4—almost always) [63]. The scale is translated and validated in the Croatian language, whereby the scale retains a high level of internal consistency (Cronbach α= 0.92) [64].

Pandemic-Specific Stressors Questionnaire for Older Adults (PSQ-OA). This was designed to measure specific stressors induced by the COVID-19 pandemic in older adults.

Three people older than 65 and two experts were involved in the construction of the questionnaire. Due to the restrictive measures implemented to isolate the general population and protect the vulnerable older population during the study, it was challenging to include a larger sample of older adults and experts in the validation process. Given the small sample of experts and older adults involved, calculating the content validity index (CVI) was not an appropriate method. However, the refinement process relied on qualitative and quantitative insights from the participants [65]. The content validation process is described below.

An initial pool of items was developed by reviewing relevant literature on stress and pandemic-specific stressors [37,66,67,68,69]. The items were modified to suit the older adult population and were informed by consultations with three older adults, with varied demographic characteristics, including differences in education, residence, gender, and age, to enhance the representativeness of the feedback about their worst fears and concerns during the pandemic. This qualitative input ensured that the questionnaire accurately reflected the real-life stressors faced by older adults during the pandemic.

Furthermore, two experts—a clinical psychologist and a master of nursing, both with extensive experience in geriatric care—evaluated the items using a 4-point Likert scale to assess their relevance, clarity, and comprehensiveness. Items with consensus ratings below 3 were revised or removed [70], resulting in adjustments to the wording of three items and the elimination of eight redundant items.

Feedback from both the experts and the older adults informed iterative revisions, ensuring that the content aligned with the real-life stressors experienced by the older adult population. Finally, after consensus was reached among the experts, the revised questionnaire was presented to the same three older adults. They assessed the items for clarity, comprehensibility, and alignment with their personal experiences. This procedure ensured that the items were both comprehensive and easily understandable, resulting in a validated instrument ready for use.

The final form of the questionnaire included items that were rated on a 5-point scale ranging from 0 (not at all) to 4 (extremely), where respondents had to assess the degree of worry to which a particular stressor refers.

Through the process of EFA analysis, two factors were extracted. The first factor, named Social Distancing Behaviours, consists of 5 items, while the second factor, Exposure to Infection, comprises 3 items. The reliability of the entire scale, measured by Cronbach’s Alpha, is 0.85. Item number 7 was omitted because its factor loading was less than 0.50. As a result, the final version of the scale consists of 8 items.

General data questionnaire. This contains data about gender, age, residence, educational/marital/employment status, and questions whether the participant lives alone/with spouse/other family members. The questionnaire also contains questions about psychiatric/neurologic diseases, as well as questions relevant to COVID-19 pandemic (e.g., source of information, availability of health services, and exposure to SARS-CoV-2 infection).

### 2.3. Statistical Analysis

This study utilized several statistical techniques to examine the relationships between pandemic-specific stressors, psychological factors, and loneliness in older adults. Data were recoded, sorted, and prepared for analysis using the SPSS version 25.0 software package.

Descriptive statistics (means and standard deviation) were used to summarize the main characteristics of the sample. Reliability analysis using Cronbach’s alpha was conducted to assess the internal consistency of the measures employed in the study. Values above 0.70 were considered indicative of acceptable reliability.

Exploratory Factor Analysis (EFA) was used to explore the underlying structure of the newly developed Pandemic-Specific Stressors Questionnaire for Older Adults (PSQ-OA). A randomized set of 289 respondents’ data was used for the calculation of relevant factors in the EFA. The goal was to identify the dimensions or factors that best represent the stressors experienced by older adults during the pandemic. This technique enables us to confirm the validity of the scale by grouping related items into factors, based on how participants responded to them. PCA extraction methods and Varimax rotation were used. Factor loadings were examined to understand the relationship between observed variables and the underlying factors. Bivariate correlations were used to test convergent validity of the PSQ-OA questionnaire and other measure used in the study, assessing the degree of association between variables that were theoretically expected to be related. Pearson correlation coefficients were calculated, with values closer to 1 or −1 indicating stronger relationships. The EFA revealed two factors, which together explained 71.3% of the total variance, demonstrating the questionnaire’s face and convergent validity.

Additionally, the remaining set of the respondents’ data (not randomized to be included in the EFA) was used to conduct confirmatory factor analysis (CFA). Confirmatory factor analysis was performed in order to assess the model fit for the Pandemic-Specific Stressors Questionnaire for Older Adults (PSQ-OA) two factor structure, identified as the Social Distancing Behaviours (Factor 1) and Exposure to infection (Factor 2). CFA measures reporting model fit are shown in the results section, including the chi square statistic, the Comparative Fit Index (CFI) [71], and the Tucker Lewis Index (TLI) [72], alongside additional fit indices such as the Standardized Root Mean Square Residual (SRMR), Goodness of Fit Index (GFI), McDonald’s Fit Index (MFI), Root Mean Square Error of Approximation (RMSEA), Hoelter’s Critical N values, and Expected Cross-Validation Index (ECVI) [73].

This method of dividing the dataset for factor analysis into subsets was conducted following Brown’s suggestions, which recommend splitting the data for exploratory factor analysis (EFA) and confirmatory factor analysis (CFA) for several reasons. EFA and CFA serve different purposes; EFA identifies the underlying structure of the data, while CFA tests whether the proposed model fits well. Using the same dataset for both analyses can lead to overfitting and inflated model fit indices, as the data would be used to both generate and confirm the model. By dividing the data, we ensured that the factor structure identified in EFA was validated on an independent subset during CFA, increasing the generalizability of the findings. Using the entire dataset for both analyses could result in CFA merely replicating the findings of EFA due to shared data characteristics rather than genuinely validating the structure [74].

Hierarchical regression analysis was conducted to determine the contribution of predictor variables (psychological factors, sociodemographic characteristics, and pandemic-specific stressors) to loneliness in older adults. This technique allows for the examination of how different sets of predictors (entered in steps) add to the explanation of the dependent variable (loneliness), adjusting for previous variables. Changes in R-squared were examined to assess the additional variance explained by each predictor.

Mediation analysis was employed to examine whether emotional stability mediated the relationship between pandemic stressors (social distancing, worry about infection) and loneliness. The study used a bootstrapping method to assess this mediation since bootstrapping (which is a resampling technique with replacement) is considered superior to other mediation methods [75,76]. Thus, for the mediation analyses, the 95% confidence intervals for the standardized indirect effects were calculated with 5000 bootstrapped resamples, and the magnitude of the standardized indirect effects was examined. Mediation analysis tests if the effect of an independent variable (e.g., pandemic stressors) on a dependent variable (loneliness) is transmitted through a mediator variable (emotional stability). This involved testing the direct effect of the independent variables (Social Distancing Behaviours and Exposure to Infection) on the dependent variable (Loneliness), as well as the indirect effect through the proposed mediator variable (Emotional Stability). The total effect, comprising both direct and indirect effects, was also examined to understand the overall relationship between variables. The significance level was set at 0.05.

## 3. Results

As mentioned before, the primary objective of this study was to identify psychological and pandemic-related stressors, along with demographic characteristics, that predict loneliness in older adults during the pandemic. In order to utilize the most appropriate research tool for this population, we developed and validated a questionnaire specifically designed to assess pandemic-specific stressors in older adults, which represented the secondary aim of the study. Therefore, the results section first presents the validation findings, followed by regression and mediation analyses exploring the contribution of different predictors to loneliness in older adults during the pandemic.

### 3.1. Validation Study

The validation study was carried out to establish the validity and internal consistency of the PSQ-OA in a cross-sectional study of 578 older adults, using EFA to test construct validity and Cronbach α to measure reliability. Convergent validity was established based on the correlation of the PSQ-OA scale with the measures often used for assessing personality and psychological states, such as TIPI, CORE, and UCLA.

### 3.2. Factor Validity

The results of the EFA are shown in Table 2. The Kaiser–Meyer–Olkin statistic proved the satisfying sampling adequacy of the data (KMO = 0.87), enabling the factor analysis. Bartlett’s test of sphericity was significant (χ^2^ (28) = 1390.04, *p* < 0.001).

In order to identify the preliminary factor structure, we retained items with a factor loading of 0.50 or above, as suggested by Hair et al. (2009) [77]. The results indicated eight items with a factor loading equal to or greater than 0.50, corresponding to two factors, a first with five and a second with three items. One item was excluded from the questionnaire due to low factor loadings.

In this way, the following two factor labels were identified, providing evidence of factor validity for PSQ-OA: Social Distancing Behaviours (Factor 1) and Exposure to Infection (Factor 2). These two factors accounted for 59.7% and 11.6% of the total variance, respectively, and together explained 71.3% of the total variance.

Confirmatory factor analysis was performed in order to assess the model fit, resulting in a significant chi square statistic, indicating a lack of fit (χ^2^ = 74,190; *p* < 0.001). Since the CFA was performed in a relatively large sample, it should be interpreted along with other measures of model fit, since using the chi-square statistic as a model fit index is sensitive to sample size (with larger sample sizes decreasing the *p*-value). The Comparative Fit Index (CFI) revealed a good model fit since it was 0.940, and the Tucker Lewis Index (TLI) was reported to be 0.911, also indicating a good model fit. The results indicate strong factor loadings for all items (ranging from 0.52 to 0.85), demonstrating that the observed variables significantly contribute to their respective factors (Table 3). The model also shows a good fit based on other fit indices, including SRMR (0.065), GFI (0.983), Hoelter’s Critical N values (118.421 at α = 0.05 and 141.978 at α = 0.01), and ECVI (0.430), indicating that the model is likely to work well with new data. The MFI (0.909) suggests that the model is a good fit when considering its complexity. Although the RMSEA (0.100) is slightly higher than the ideal range, it is still within an acceptable range, implying that there may be some room for improvement to enhance the fit. Taken together, these indices support the conclusion that the questionnaire is valid, with robust psychometric properties for measuring pandemic-specific stressors in older adults. While RMSEA (0.100) indicates marginal fit, the strength of the other indices suggests that the model overall is reasonable and fit for use. This conclusion relies on the finding by McNeish et al., which states that a higher RMSEA may still indicate acceptable model fit (if other indicators supports good validity) [78].

In short, the results of EFA and CFA confirm the two-factor structure of the questionnaire. While Factor 1 captures a large portion of the variance, the 11.6% contribution from Factor 2 is not negligible and indicates meaningful differentiation. Further, the two identified factors are conceptually distinct. Next, the factor loadings clearly align with two separate latent constructs. Each factor demonstrates strong internal consistency, as shown by high loadings (>0.5). The CFI (0.940) and TLI (0.911) both indicate a good model fit, meaning that the two-factor model (Social Distancing and Exposure to Infection) explains the data well. Although the chi-square statistic is significant (χ^2^ = 74.190, *p* < 0.001), this result is expected in larger samples due to the chi-square test’s sensitivity to sample size. In this case, the CFI and TLI provide more reliable indicators of model fit, showing that despite the significant chi-square, the model is indeed a good fit for the data.

### 3.3. Reliability

Table 4 displays the Cronbach’s alpha coefficient if items are excluded. The overall Cronbach alpha was 0.877 (C.I. 0.861–0.891).

### 3.4. Convergent Validity

Table 5 shows the correlations of the PSQ-OA with the measures of emotional stability, psychological distress, and loneliness. As can be seen, almost all correlations were significant (*p* < 0.001), especially with the CORE and UCLA scales. These findings support the convergent validity of the PSQ-OA.

### 3.5. Factors Contributing to Loneliness Among Older Adults During the Pandemic

In line with the next research aim, we examined whether personal characteristics (sociodemographic factors, emotional stability, psychological distress, as well as pandemic-specific stressors) could alter the levels of loneliness in older adults during the pandemic.

Accordingly, regression analyses were conducted where sociodemographic characteristics (gender, age, education, employment), levels of exposure to pandemic-specific stressors, and levels of psychological distress were included as predictor variables, while level of loneliness was a criterion variable (β = −0.285, *p* < 0.001). As presented in Table 6, in the first step, sociodemographic factors (age, gender, and residence, as well as marital, educational, and employment status) together explained 7.912% of the variance of loneliness. Here, only marital status was a significant predictor of loneliness, indicating that older people who were married had lower levels of loneliness compared to people who were widowed, divorced, or never married. When the variables of psychological distress and emotional stability are added in the model in the second step, it is evident that the percentage of explained variance increases to 34%, where both emotional stability (β = −0.139, *p* < 0.001) and psychological distress (including problems β = 0.207, *p* < 0.001, life functional difficulties β = 0.241, *p* < 0.001, and risky behaviours β = 0.141, *p* < 0.001) significantly contributed to explaining the loneliness of older adults during the pandemic.

Finally, by adding the contribution of the pandemic-specific stressors in the third step (β = 0.109, *p* = 0.005), the total percentage of explained variance in loneliness was 35.2%.

In short, older adults who were married had a higher degree of emotional stability and fewer psychological problems, showed less risky behaviours and functional difficulties, and had a lower degree of loneliness during the pandemic. In this way, the mentioned factors serve as protective factors that reduce the experience of loneliness among older adults during the pandemic.

### 3.6. The Mediating Effects of Emotional Stability Between Experiencing Pandemic-Specific Stressors and Loneliness

In order to explore the potential mediating role of emotional stability between levels of experiencing pandemic-specific stressors and loneliness, a mediation analysis was conducted (Table 7). Within this analysis, the total scores of pandemic-specific stressors on the subscales Social Distancing and Exposure of Infection were used as predictors, with emotional stability as a mediator and loneliness as a criterion variable. The obtained results showed a significant direct effect of social distancing behaviours on loneliness, with a coefficient of 0.317 (*p* < 0.001). Emotional stability also significantly mediates this relationship, with an indirect effect of 0.065 (*p* = 0.002). The total effect of social distancing behaviours on loneliness is 0.382 (*p* < 0.001), which includes both direct and indirect effects. No significant direct effect is found between exposure to infection and loneliness (*p* = 0.672). Additionally, emotional stability does not mediate the relationship between exposure to infection and loneliness, as both the direct and indirect effects are non-significant (*p* > 0.05). The total effect is also insignificant.

The results suggest that emotional stability helps reduce loneliness in older adults affected by social distancing behaviours, but it does not appear to mediate the relationship between exposure to infection and loneliness. This finding highlights the importance of emotional stability in coping with stressors related to the pandemic, especially in terms of social distancing, while emphasizing that the fear of infection does not have the same mediated impact on loneliness. This model suggests that some of the effect of Social Distancing was translated through the negative effect of emotional stability on loneliness. In other words, higher levels of stress from Social Distancing reduced emotional stability, and this in turn increased loneliness in participants.

## 4. Discussion

This study provides the first measurement tool designed to examine pandemic-specific stressors in older adults. The exploratory factor analysis (EFA) of the PSQ-OA revealed two distinct factors, “Social Distancing Behaviours” and “Exposure to Infection”, where these factors together explained 71.3% of the total variance. The results of the confirmatory factor analysis indicate a good model fit. The high correlation between the items and the total score, along with the high Cronbach alpha coefficients, suggests that each item contributes to measuring the same construct, and the scale as a whole reliably measures pandemic-specific stressors. Further, the PSQ-OA has significant correlations with emotional stability and psychological distress and loneliness, particularly with the CORE and UCLA scales, suggesting that the PSQ-OA is effectively measuring constructs that are theoretically related to pandemic-specific stress, thus supporting its convergent validity.

The proposed regression model shows that older adults experienced lower levels of loneliness during the pandemic when they were married, exhibited higher emotional stability, encountered fewer psychological problems and pandemic-specific stressors, engaged in less risky behaviours, and demonstrated better daily functionality. Although the R^2^ value of 7.9% in the first step is relatively low, marital status was still identified as a significant predictor of loneliness, suggesting that being married is associated with lower levels of loneliness. The limited R^2^ reflects the contribution of all socio-demographic factors together, not just marital status. Despite this, marital status remains an important factor in other steps, as it directly influences loneliness, with married individuals reporting less loneliness than those who are widowed, divorced, or never married, as confirmed in other research [79]. The model’s explanatory power increases when additional psychological factors are included, but marital status continues to be a meaningful predictor.

The results suggest that psychological factors, followed by marital status, are significant contributors to loneliness among older adults, while pandemic-specific stressors contribute only modestly to the overall effect. This study aligns with previous research, which consistently highlights the protective effect of co-habitation against loneliness among older adults during the pandemic [14,24,80]. We also found that the inclusion of the PSQ-OA to the regression model resulted in a modest increase in explained variance. However, we consider that the PSQ-OA remains a valuable tool. The scale was specifically designed to capture stressors unique to pandemic conditions, offering insights beyond general stress and psychological factors. The PSQ-OA can help identify pandemic-specific stressors affecting older adults, enabling a deeper understanding of how such stressors impact mental health and well-being in this population. Furthermore, this instrument is relevant for future health crises, as it allows researchers and practitioners to explore a range of outcomes beyond loneliness, including anxiety, resilience, and coping.

However, no research was found to assert that psychological factors predominantly influenced the loneliness of older adults during the pandemic, compared to sociodemographic and pandemic-specific stressors. The results of the mediation analysis suggest that emotional stability might play an important role in reducing loneliness among older adults, particularly in the context of social distancing behaviours during the pandemic. Older adults who experienced greater social distancing behaviours felt lonelier, and this effect was stronger among those with lower emotional stability. This finding supports the notion that emotional stability, as a stable personality trait, can help older adults cope with the stressors of social distancing by employing adaptive coping strategies, maintaining virtual social connections, and engaging in meaningful solitary activities. On the other hand, the mediating effect of emotional stability between fear of infection and loneliness was not established, which suggests the overall limited applicability of this pathway. One possible explanation is that exposure to infection may be less important in explaining loneliness in older adults because emotional and social factors, such as emotional stability and social support, tend to have a stronger influence on reducing feelings of isolation. Furthermore, older adults may place more emphasis on safety measures and health concerns, focusing on behaviours like social distancing rather than the actual risk of infection, which could lessen the impact of perceived exposure. Future research should explore other potential mediators and consider additional factors that might affect loneliness during the pandemic.

Although previous meta-analyses [81] have found associations between emotional stability and loneliness, no studies specifically examined this relationship in the context of the pandemic. This study’s findings contribute novel insights into how emotional stability and psychological distress interact with loneliness in older adults during the COVID-19 pandemic. Although the significance of pandemic-specific stressors measured by the PSQ-OA questionnaire on loneliness in older adults is relatively small, it does not prevent its use in other research fields.

Pandemic-specific studies have also found that stressors like fear of infection, depression, and social isolation due to lockdowns did increase feelings of loneliness among older adults [82]. Surprisingly, the additional variance explained by these factors in this study was relatively small, consistent with the modest increase from 34% to 35%. This modest increase in the variance explained by pandemic-specific stressors could indicate that while these factors did contribute to increased loneliness among older adults, their impact was relatively minor, but still significant, compared to other persistent and pre-existing factors contributing to loneliness in older people.

The study’s results provide essential insights into addressing loneliness and psychological distress among older adults during crises and have practical implications. While the findings may not fully apply to all future crises due to varying stressors and contexts, lessons from the COVID-19 pandemic offer valuable insights for timely support to older adults in future emergencies. Prioritizing psychological well-being, emotional stability, and social connections can help prevent loneliness and reduce isolation’s negative effects in older adults. The PSQ-OA questionnaire, although showing a small impact of pandemic-specific stressors on loneliness, can be used to assess social isolation and fear, linking them to broader mental health issues like anxiety and depression. Mental health professionals, healthcare providers, policymakers, caregivers, and community organizations can use these findings to enhance support for older adults by focusing on mental well-being and reducing isolation. These groups can help mitigate loneliness and its psychological impacts in future emergencies.

## 5. Limitations

However, the study has some limitations. First, the use of the snowball sampling, which provided access to a hard-to-reach older population by recruiting participants through referrals, could also have led to sampling bias, making the sample non-representative and heavily influenced by the initial subjects (students). The snowball sampling method may have led to a sample of older adults with similar backgrounds and experiences, over-representing socially connected individuals and underrepresenting isolated ones. Further, the sample depended heavily on social networks, which could have hindered the recruitment if participants had limited or homogeneous networks. This could skew results and limit the ability to generalize the findings to the broader population, especially those from different regions or with diverse social contexts. Additionally, the biases of the students selecting participants may have influenced the sample, potentially underestimating the impact of pandemic stressors on more isolated or distressed individuals. Additionally, ensuring privacy and controlling the sample was challenging, and data quality might have varied due to participants’ willingness for participation and their relationship with the researcher.

To avoid biases in future studies, researchers can use more random or stratified sampling methods to ensure a more representative sample that reflects the broader population of older adults, including those from diverse social backgrounds and levels of engagement. Additionally, reaching out to community organizations or healthcare providers to recruit participants from various social and demographic groups can help mitigate the over-representation of socially connected individuals and ensure a more accurate understanding of pandemic-specific stressors and loneliness. Given the questionnaire validation process, we acknowledge that certain dimensions of construct validity, such as discriminant validity, could further enhance the questionnaire’s validity. Therefore, we recommend that such additional measures be included in future studies. Finally, although test–retest reliability is undoubtedly an important indicator of the temporal stability of a questionnaire, in this case this was not feasible given that it was a cross-sectional study conducted during the peak of the pandemic on older adults. The circumstances at the time made it significantly more difficult to conduct the data collection process and the possible implementation of the longitudinal research necessary for test–retest analysis, which we also recommend to researchers in future studies.

## 6. Conclusions

In conclusion, the findings highlight the critical role of psychological factors, particularly emotional stability, in mitigating loneliness when facing pandemic-specific stressors, alongside the minor but relevant impact of sociodemographic factors, such as marital status. By identifying these key predictors of loneliness, this study underscores the importance of targeted interventions that address psychological well-being and social support for older adults during crises. In the event of similar public health crises, these research results should certainly be considered. This study developed and validated the Pandemic-Specific Stressors Questionnaire for Older Adults (PSQ-OA), offering a reliable tool for assessing unique stressors faced by older adults during crises. Although the PSQ-OA shows a modest contribution to explaining loneliness, it remains a valuable tool for identifying pandemic-specific challenges that may contribute to psychological distress in older adults. The PSQ-OA equips practitioners and policymakers with an effective instrument for identifying at risk individuals and implementing timely, supportive measures to protect the mental health of older adults in future crises. It is recommended that clinicians and policymakers use these findings to determine the best interventions to address the mental health and well-being of older people.

## Figures and Tables

**Table 1:** Sociodemographic characteristics (N = 578).

Sociodemographic Characteristics	N	%	M	SD	Range
Age			74.09	6.72	65–97
Gender					
Male	225	38.9			
Female	353	61.1			
Place of residence					
Urban	388	67.1			
Rural	190	32.9			
Marital status					
Married	343	59.3			
Divorced	24	4.2			
Never married	34	5.9			
Widowed	177	30.6			
Employment status					
Retired	479	82.9			
Never employed	72	12.5			
Worked full-time	9	1.6			
Worked part-time	17	2.9			
Completed education					
No school	14	2.4			
Uncompleted primary school	53	9.2			
Primary school	134	23.2			
High school	257	44.5			
College/Bachelor’s degree	58	10.0			
Faculty/Master’s degree	54	9.3			
Doctorate/Postgraduate	8	1.4			

**Table 2:** Factor analysis of the PSQ-OA (EFA performed on a randomized sample, N = 289).

	2-Factor Solution	
	Factor 1 Social Distancing Behaviours	Factor 2 Exposure to Infection
Kaiser–Meyer–Olkin Measure of Sampling Adequacy	0.87	
Bartlett’s test of sphericity	χ^2^ (28) = 1390.04, *p* < 0.001	
Initial Eigenvalues	4.78	0.92
% of Variance	59.69	11.55
Cumulative %	59.69	71.25
Rotation Sums of Squared	2.94	2.76
% of Variance	36.73	34.51
Cumulative %	36.73	71.25
Item loadings		
Uncertainty about the duration of the pandemic		0.78
Fear of infection for my loved ones.		0.83
Isolation from family members and/or other people	0.82	
Loneliness due to less frequent socializing with friends and family	0.86	
Inability to go outside	0.87	
Fear that others will avoid me if I get sick	0.55	
I fear that healthcare will be denied to me because of the pandemic	0.50	
Fear of getting infected		0.78

Note: Total variance explained and factor component loadings for the PSQ-OA. Abbreviations: χ^2^: chi-square; PSQ-OA: Pandemic-Specific Stressors Questionnaire for Older Adults.

**Table 3:** Parameter estimates for the CFA (performed on a randomized sample, N = 289).

Factor	Indicator	Estimate	SE	Z	*p*	Lower 95% CI	Upper 95% CI	Standardized Factor Loadings
Factor 1	Isolation from family members and/or other people.	1.092	0.076	14.409	<0.001	0.944	1.241	0.75
	Loneliness due to less frequent socializing with friends and family.	1.114	0.070	15.878	<0.001	0.977	1.252	0.78
	Inability to go outside.	1.028	0.072	14.356	<0.001	0.888	1.169	0.72
	Fear that others will avoid me if I get sick.	0.700	0.087	8.066	<0.001	0.530	0.870	0.55
	I fear that healthcare will be denied to me because of the pandemic.	0.643	0.083	7.733	<0.001	0.480	0.806	0.52
Factor 2	Uncertainty about the duration of the pandemic.	0.975	0.065	14.970	<0.001	0.847	1.103	0.80
	Fear of infection for my loved ones.	1.152	0.066	17.386	<0.001	1.022	1.282	0.85
	Fear of getting infected.	0.940	0.077	12.187	<0.001	0.789	1.091	0.72

Abbreviations: Cl: Confidence Level; SE: Standard error; Z: z-value; *p*: *p*-value.

**Table 4:** Reliability measures of the PSQ-OA.

Items	Mean	SD	Squared Multiple Correlation	Cronbach’s Alpha if Item Deleted
Uncertainty about the duration of the pandemic.	3.61	1.22	0.48	0.87
Fear of infection for my loved ones.	3.88	1.27	0.56	0.86
Isolation from family members and/or other people.	2.89	1.42	0.57	0.85
Loneliness due to less frequent socializing with friends and family.	2.95	1.35	0.64	0.86
Inability to go outside.	2.67	1.37	0.57	0.86
Fear that others will avoid me if I get sick.	2.38	1.45	0.38	0.87
I fear that healthcare will be denied to me because of the pandemic.	2.63	1.44	0.39	0.87
Fear of getting infected.	3.31	1.44	0.53	0.86

Abbreviations: PSQ-OA: Pandemic-Specific Stressors Questionnaire for Older Adults.

**Table 5:** Tests of convergent validity: Correlations of the PSQ-OA with TIPI-ES, CORE, and UCLA.

	PSQ-OA _Total	PSQ-OA_EI	PSQ-OA_SD	TIPI_ES	CORE_SW	CORE_P	CORE_F	CORE_R	CORE _Total	UCLA _Total
PSQ-OA _total	-	0.85 **	0.95 **	−0.16 **	0.32 **	0.39 **	0.34 **	0.23 **	0.40 **	0.30 **
PSQ-OA _Exposure to infection	0.85 **	-	0.63 **	−0.09 *	0.16 **	0.22 **	0.11 **	0.13 **	0.19 **	0.19 **
PSQ-OA _Social distancing behaviours	0.95 **	0.63 **	-	−0.17 **	0.38 **	0.44 **	0.43 **	0.26 **	0.47 **	0.32 **

Note: * *p* < 0.05; ** *p* < 0.01; Abbreviations: PSQ-OA: Pandemic-Specific Stressors Questionnaire for Older Adults; CORE: Clinical Outcomes in Routine Evaluation; UCLA: revised UCLA Loneliness Scale; PSQ-OA_EI: Exposure to infection; PSQ-OA_SD: Social distancing behaviours; TIPI_ES: Ten-Item Personality Inventory Emotional Stability; CORE_SW: Subjective Well-being; CORE_P: Problems/Symptoms; CORE_F: Life functioning difficulties; CORE_R: Risky Behaviours. The CORE-SW and CORE-F were scored in the negative direction.

**Table 6:** Results of hierarchical regression analyses using levels of loneliness as criteria.

	Predictors	B	β	t	*p*
Step 1	(Constant)	17.088		5.875	0.000
	Gender	0.295	0.026	0.593	0.553
	Marital status	−3.225	−0.285 **	−6.304	0.000
	Place of residence (urban/rural)	0.786	0.066	1.611	0.108
	Age	−0.003	−0.004	−0.082	0.934
	Education	−1.099	−0.080	−1.934	0.054
	Employment	0.446	0.028	0.649	0.516
	R	0.281			
	R^2^	0.079			
	F	8.163 **			
	df	576			
Step 2	(Constant)	16.564		6.333	0.000
	Gender	0.234	0.021	0.550	0.582
	Marital status	−2.156	−0.190 **	−4.873	0.000
	Place of residence (urban/rural)	0.426	0.036	1.021	0.308
	Age	−0.019	−0.023	−0.614	0.540
	Education	−0.767	−0.056	−1.587	0.113
	Employment	0.733	0.046	1.254	0.211
	Emotional Stability	−0.522	−0.139 **	−3.615	0.000
	Subjective wellbeing	−0.123	−0.067	−1.110	0.268
	Problems symptoms	0.135	0.207 **	3.701	0.000
	Life functioning difficulties	0.185	0.241 **	4.051	0.000
	Risky Behaviours	0.296	0.141 **	3.539	0.000
	R	0.586			
	R^2^	0.343			
	F	26.823 **			
	df	576			
Step 3	(Constant)	16.051		6.161	0.000
	Gender	0.384	0.034	0.902	0.367
	Marital status	−2.169	−0.192 **	−4.933	0.000
	Place of residence (urban/rural)	0.197	0.017	0.468	0.640
	Age	−0.031	−0.038	−1.014	0.311
	Education	−0.697	−0.051	−1.451	0.147
	Employment	0.776	0.049	1.334	0.183
	Emotional Stability	−0.521	−0.138 **	−3.629	0.000
	Subjective wellbeing	−0.122	−0.067	−1.110	0.267
	Problems symptoms	0.116	0.178 **	3.139	0.002
	Life functioning difficulties	0.177	0.230 **	3.885	0.000
	Risky Behaviours	0.287	0.137 **	3.444	0.001
	PSQ-OA	0.076	0.109 **	2.845	0.005
	R	0.594			
	R^2^	0.352			
	F	25.572 **			
	df	576			

Note: ** *p* < 0.01; Abbreviations: B: unstandardized regression coefficient; β: standardized regression coefficient; t: *t*-test; R: multiple regression coefficient; R^2^: variance explained by the predictors; F: F-ratio; df: degree of freedom; PSQ-OA: Pandemic-Specific Stressors Questionnaire for Older Adults.

**Table 7:** Mediating role of emotional stability between pandemic-specific stressors (Social Distancing Behaviours and Exposure to Infection) and loneliness.

	Social Distancing Behaviours (Factor 1) as Predictor			Exposure to Infection (Factor 2) as Predictor			Path Coefficients		
	Direct Effects	Indirect Effects	Total Effects	Direct Effects	Indirect Effects	Total Effects	Social Distancing Behaviours and Emotional Stability	Exposure to Infection and Emotional Stability	Emotional Stability and UCLA
Estimate	0.317	0.065	0.382	0.027	−0.004	0.023	−0.059	0.004	−1.105
SE	0.061	0.021	0.063	0.064	0.020	0.067	0.017	0.018	0.143
*p*	<0.001	0.002	<0.001	0.672	0.829	0.736	<0.001	0.829	<0.001
95% CI	0.199 to 0.443	0.023 to 0.114	0.262 to 0.512	−0.088 to 0.135	−0.052 to 0.037	−0.102 to 0.133	−0.093 to −0.024	−0.032 to 0.040	−1.386 to −0.824

Note: Criterion: UCLA loneliness; Mediator: Emotional Stability; Abbreviations: SE: Standard error; *p*: *p*-value; Cl: Confidence Level.

## Data Availability

Data are not publicly available. Researchers interested in analysing the dataset can contact the authors to discuss potential collaborations for archival analysis.
